# Long-Term Prognostic Analysis after Endoscopic Endonasal Surgery for Olfactory Neuroblastoma: A Retrospective Study of 13 Cases

**DOI:** 10.1371/journal.pone.0166046

**Published:** 2016-11-02

**Authors:** Luyao Zhang, Kai Niu, Kang Zhu, Cui Xia, Jing Yan, Wei Zhao, Junrong Wei, Maoli Duan, Guoxi Zheng

**Affiliations:** 1 Department of Otorhinolaryngology Head and Neck Surgery, the Second Affiliated Hospital of Xi'an Jiaotong University, Xi’an, Shaanxi, China; 2 Department of Clinical Science, Technology and Intervention, Karolinska Institutet, Karolinska Hospital, Stockholm, Sweden; 3 Department of Otorhinolaryngology Head and Neck Surgery, the No.1 Affiliated Hospital of Jilin University, Changchun, Jilin, China; 4 Department of Otorhinolaryngology, Shangluo Central Hospital, Shangluo, Shaanxi, China; George Washington University, UNITED STATES

## Abstract

**Objectives:**

To summarize the characteristics and long–term outcomes of olfactory neuroblastoma through the analysis of 13 cases in single institution, with the assessment of treatment modality, prognostic factors.

**Method:**

A retrospective study of thirteen cases diagnosed as olfactory neuroblastoma and underwent combined treatments during the period 2000–2010. Statistical analysis was performed to search for prognostic factors and compared different treatment modalities.

**Results:**

13 patients were enrolled in this study, including 8 male and 5 female, ranging from 15 to 69 (median 43) years old. One patient at stage A was only treated with endoscopic endonasal surgery (EES). Seven patients were treated with preoperative radiotherapy and EES, two with EES and postoperative radiotherapy, and the other three with combined radiotherapy and chemotherapy. The range of follow-up time varied from 23 to 116 months (median 65 months). The 5-year overall survival rate was 46.2% (6/13). To date, these thirteen patients have not suffered local recurrences while two patients had lymph node recurrences and one had distant metastasis in the bone marrow. In 13 patients, 61.5% were diagnosed as late T stage (T3/4), 69.2% late Kadish stage (C/D) and 53.8% were high Hyams grade (I/ II), which indicated poor prognosis. Related prognostic factors were the TNM stage (T stage P = 0.028, N stage P = 0.000, M stage P = 0.007), Kadish stage (P = 0.025) and treatment modality (P = 0.015).

**Conclusion:**

Late stage of TNM and Kadish staging system indicated a poor prognosis. Combined treatment modality, including endoscopic endonasal surgery, achieved a better outcome than non-surgical approach.

## Introduction

Olfactory neuroblastoma (ONB), also known as esthesioneuroblastoma (ENB), is a rare malignant neoplasm arising from the upper nasal cavity. It was originally described by Berger and colleagues in 1924 using the term “esthesioneuroepitheliome olfactif” [1]. It represents approximately 3% of malignant sinonasal tumors and the incidence has been estimated at 0.4 per million [2]. The common clinical symptoms are nonspecific sinonasal complaints, including unilateral nasal obstruction, recurrent epistaxis, visual disturbances, headache and others. Kadish and co-workers proposed a classification system of stage A, B, and C based on their experience of 17 cases[3], without consideration of metastasis. To make up for the insufficiency of Kadish stage system, Foote and Morita added stage D (cervical nodal or distant metastasis) to the Kadish classification [4]. In 1992, Dulguerov and Calcetta proposed a tumor, node and metastasis (TNM) staging system, largely based on computed tomography(CT) and magnetic resonance imaging (MRI) scans, allowing for cervical lymph node involvement and distant metastasis [5]. These tumors have a tendency towards local invasion (commonly orbit and anterior skull base) and distant metastases (commonly neck, lungs, liver and bones). Patients with ONB are diagnosed definitively by Hematoxylin-eosin (H-E) and immunohistochemical (IHC) staining. Hyams developed a histopathologic grading system, taking all of the histopathological features into account. While there is controversy and divergency in the treatment modality, it typically involves surgery and radiotherapy, with or without chemotherapy[6]. To better understand the clinical features of ONB, We retrospectively analyzed our cases over last ten years to summarize the characteristics and prognostic factors. Additionally, we performed a systematic review of previous published studies to compare our findings with them.

## Materials and Methods

### Ethics Statement

This study was conducted under the supervision and approval of the Medical Ethics Committee of the Second Affiliated Hospital of Xi'an Jiaotong University (Permit Number: 2015–020). These selected patients or their family members in this study have given written informed consents to publish these case details.

### Patients recruitment

We performed a retrospective study of patients diagnosed with ONB who were treated in our otolaryngology department from 2000 to 2010. As a result, this study included a cohort of 13 cases, who went to ENT or oncology out-patient department initially and then were transferred to ENT in-patient department to received hospital treatment.

### Information collection

We collected detailed clinical data regarding age, gender, TNM stage, modified Kadish stage, Hyams grade and treatment modality individually, and then telephoned the patients or their family members to obtain follow-up data. Pathological slices of all patients were reviewed to summarize the common features of ONB and to confirm the Hyams grade. The clinical therapeutic effect was demonstrated by the contrast of CT or MRI scans before and after treatment.

### Statistical analysis

Overall survival rate was defined as the time from initial treatment to death for whatever reasons and was calculated based on the Kaplan- Meier method, while univariate analysis was carried out by using the log-rank test. A ‘p’ value < 0.05 was considered significant. Statistical analysis was performed with SPSS 22.0 (IBM Corp.).

## Results

### Patient Demographics and clinical symptoms

Thirteen patients were recruited in this study, 8 men and 5 women, ranging from 15 to 69 (median 43) years old. The initial clinical symptoms were unilateral nasal obstruction (10/13), epistaxis (6/13), visual disturbance (3/13), headache (2/13) and others. Follow-up was accomplished with all patients, with median duration of 65 months ranging from 23 to 116 months.

### Distribution of staging and grading

The classification of the 13 cases with respect to Dulguerov TNM staging, modified Kadish staging and Hyams grading was calculated. As for the TNM staging system, three patients were diagnosed at stage T1, two at stage T2, six at stage T3 and two at stage T4. Moreover, three patients had cervical lymph node metastasis (N1 stage) and two had distant metastasis present (M1 stage). According to the Kadish classification, one patient was at stage A, three were at stage B, six were at stage C and three were at stage D. Within the six patients at stage C, four had invasion of the cribriform plate, one had partial lysis of the anterior skull base and another had a spread into the bone orbit. Moreover, three patients at stage D consisted of three neck lymphatic metastases and two bone metastases. With regard to Hyams grading classification, one patient was at grade Ⅰ, five were at grade Ⅱ and the rest of seven were at grade Ⅲ.

### Treatment modality

Treatment modality was determined for each patient individually, allowing for suggestion of the surgeon and decision of the patient. The patient at stage A only underwent endoscopic endonasal resection. Seven patients, including two at stage B, four at stage C and one at stage D, received preoperative external beam radiation with 55 Gy to decrease the tumor volume and insure the tumor resectable. Two patients (one at stage B and the other at stage C) had positive microscopic margins and received postoperative external beam radiation with 60 Gy. Three patients who were at stage C or D underwent both radiotherapy of 65Gy and chemotherapy of etoposide and cisplatinum, and two of them also underwent a modified neck dissection for excision of the metastatic cervical lymph nodes. Patient demographics, tumor characteristics and treatment modality are summarized in Table 1.

**Table 1:** Patient demographics, tumor characteristics, and treatment modality.

Patient no.	Age (years)	Gender	TNM stage	Kadish stage	Hyams grade	Treatment protocol	Final status	Follow-up (months)
1	48	F	T3N0M0	C	Ⅱ	R+S	Died	56
2	27	M	T2N0M0	B	Ⅱ	R+S	Alive	104+
3	36	M	T3N0M0	C	Ⅲ	R+C	Died	65
4	43	M	T3N0M0	C	Ⅲ	R+EES	Alive	52+
5	24	F	T3N0M0	C	Ⅱ	R+EES	Died	89
6	54	M	T4N1M1	D	Ⅲ	R+C+D	Died	29
7	63	F	T3N0M0	C	Ⅱ	R+EES	Alive	116+
8	15	M	T1N0M0	B	Ⅱ	R+EES	Alive	63+
9	58	M	T1N0M0	A	Ⅰ	EES	Alive	90+
10	33	F	T3N1M0	D	Ⅲ	R+EES+D	Died	23
11	38	F	T1N0M0	B	Ⅲ	EES+R	Alive	109+
12	47	M	T2N0M0	C	Ⅲ	EES+R	Died	91
13	69	M	T4N1M1	D	Ⅲ	R+C	Died	46

Abbreviation: F: female; M: male; EES: endoscopic endonasal surgery; R: radiotherapy; C: chemotherapy; D: neck dissection.

### Imaging features

**Fig 1:**
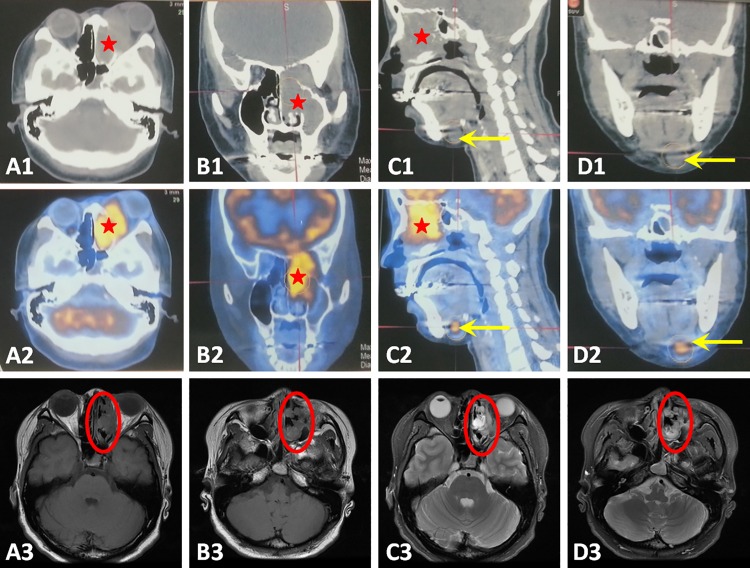
CT scans, PET scans and MRI findings of the tumor and metastatic cervical lymph nodes. The tumor was seen in the left nasal cavity, maxillary and ethmoidal sinus on CT scans (A1, B1and C1, red stars) and PET scans (A2, B2 and C2, red stars). MRI T1-weighted image showed a low-intense mass (A3 and B3, red circles) while T2-weighted image showed a relatively high-intense mass (C3 and D3, red circles). Metastatic cervical lymph nodes can been seen on CT scans (C1 and D1, yellow arrows) but more prominent on the PET scans (C2 and D2, yellow arrows).

**Fig 2:**
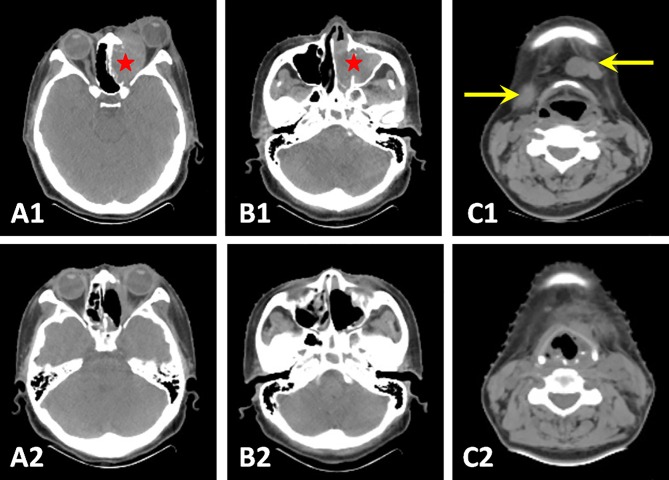
Pre- and post-operative CT scans. Endoscopic surgery released intraorbital compression behind the left eyeball (A1 and A2, red star) and resected tumor mass in left nasal cavity, maxillary and ethmoidal sinus (A2, B1and B2). Elective neck dissection removed those metastatic cervical lymph nodes (C1 and C2, yellow arrows). Those post-operative CT scans were taken after 2 months of surgery.

Local extension of the tumor is evaluated using radiological examination. CT scan is the initial radiological choice because of a better display of tumor size and bony erosion (Fig 1). ONB typically shows a relatively homogeneous soft-tissue mass in the nasal cavity, leading to a non-specific radiological appearance. Positron emission tomography (PET) is not a routine evaluation but can be used as an adjunct to CT, clearly showing cervical lymph node metastasis (Fig 1). In order to distinguish tumor tissue from nasal secretions, MRI is also required. ONB appears as low-intense to grey matter on the T1-weighted image and relatively high-intense on the T2-weighted image (Fig 1). CT scan was also adopted in the follow-up study to compare pre-and post-operative effect (Fig 2).

### Histopathology characteristics

**Fig 3:**
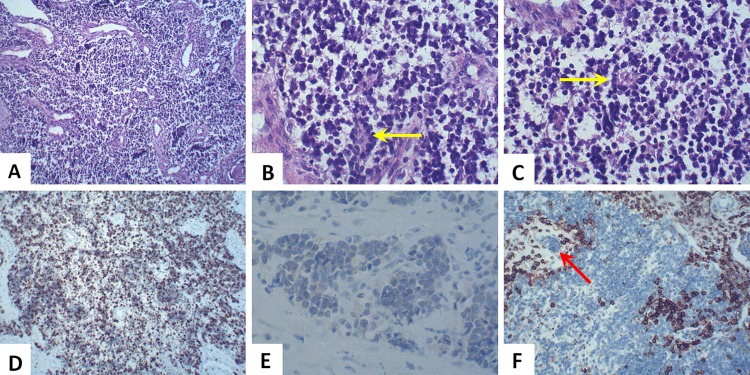
Histopathology characteristics of ONB. H-E staining revealed a nest-like or cord-like tumor mass, showing uniform small cells with prominent round nuclei and eosinophilic fibrillary background (A, ×100). Pseudorosette formation (Homer-Wright rosettes) consisted of a ring of columnar cells and the presence of fibrillary material within the central space (B and C, yellow arrow, ×400). IHC staining for Ki-67 was 60% positive in all neoplastic cells (D, ×100) while staining for NSE was mild positive (E, ×400). LCA (CD45) was negative in ONB tissue but positive in lymphoid tissue (F, ×100). Tumor cells were metastatic into the lymphatic vessel (F, red arrow, ×100).

All patients were diagnosed as ONB through histopathological analysis of postoperative specimens or the biopsy of lesion. Typical light microscopy features of Hematoxylin-eosin (H-E) staining were as follows: homogeneous small cells with uniform round nuclei, with rosette or pseudorosette formation, and eosinophilic fibrillary intercellular background material. The neoplastic cells have prominent nuclei, and the cell borders are difficult to see (Fig 3A, 3B and 3C). In the case of difficulty to differentiate ONB from other small cell malignant tumors, immunohistochemistry (IHC) staining is very important. As for IHC, Ki-67, neuron specific enolase (NSE), synaptophysin, chromogranin and S-100 protein were often positive to various degrees; while cytokeratin, vimentin and leucocytic common antigen (LCA) were usually negative (Fig 3D, 3E and 3F).

### Prognostic factors associated with Overall Survival

**Fig 4:**
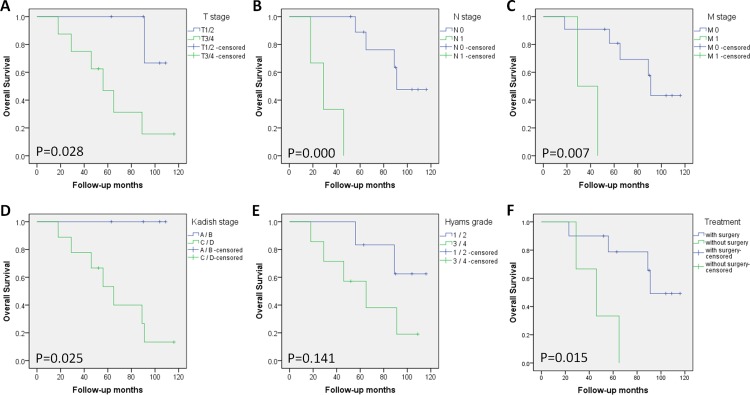
Kaplan-Meier survival analysis of the overall survival, using Log-rank test. Survival curves were plotted based on potential prognostic factors respectively, which were T stage (A), N stage (B), M stage (C), modified Kadish stage (D), Hyams grade (E) and treatment modality (F). P values were as shown in each figure.

All patients had regular follow-ups, with a median duration of 65 months, ranging from 23 to 116 months (Table 1). The 5-year overall survival (OS) was 46.2% (6/13). Patient characteristics–age and gender, tumor features and treatment modalities were evaluated with respect to OS by univariate analysis. Analysis revealed that age (log-rank p = 0.727), gender (log rank p = 0.972) and Hyams grading (log rank p = 0.141) had no effect on OS. However, T stage (log-rank p = 0.028), N stage (log-rank p = 0.000), M stage (log-rank p = 0.007), modified Kadish stage (log-rank p = 0.025), and treatment modality (log-rank p = 0.015) indeed affected OS with statistical differences (Table 2). Survival curves were plotted according to those potential prognostic factors (Fig 4).

**Table 2:** Follow-up and overall survival rates (OS).

Variables	Number (%)	5-year OS number (%)	Log-rank test	
			Χ^2^	P value*
Age (years)				
<40	6(46.2)	3(50.0)	0.122	0.727
≥40	7(53.8)	3(42.9)		
Gender				
Male	8(61.5)	4(50.0)	0.001	0.972
Female	5(38.5)	2(40.0)		
TNM stage				
T1/2	5(38.6)	4(80.0)	4.810	0.028 ^a^
T3/4	8(61.5)	2(25.0)		
N0	10(76.9)	6(60.0)	15.809	0.000 ^b^
N1	3(23.1)	0(0)		
M0	11(84.6)	6(54.6)	7.175	0.007 ^b^
M1	2(15.4)	0(0)		
Kadish stage				
A/B	4(30.8)	4(100.0)	4.993	0.025 ^a^
C/D	9(69.2)	2(22.2)		
Hyams grade				
Ⅰ/Ⅱ	6(46.2)	4(66.7)	2.168	0.141
Ⅲ/Ⅳ	7(53.8)	2(28.6)		
Treatment				
With EES	9(69.2)	6(66.7)	5.906	0.015 ^a^
Without EES	4(30.8)	0(0)		

* P values were calculated using Log-rank test.

a: P<0.05

b: P<0.01.

## Discussion

Olfactory neuroblastoma (ONB) is an uncommon malignant neoplasm in nasal cavity and paranasal sinuses, with a tendency to invade the bony orbit or anterior skull base. Although Trojanowski JQ demonstrated the presence of neurofilament proteins (NFP) in ONB tissue to reveal its neural origin in 1982[7], the exact origin still remains unclear for lack of direct evidence of the olfactory epithelium[6]. Due to its rarity, it is difficult to summarize the characteristics and prognostic factors of the disease. Age distribution was uniform in this study with a median of 43years old. Song C and colleagues reported a bi-peak incidence at the second and sixth decades of life [8], but majority studies supported a unimodal peak in the fourth and fifth decades of life [9, 10]. To date, no clear aetiological agent has been demonstrated in human beings [6]. In ONB, a complex pattern of gene alterations were described, for instance, gains in 20q and 13q, nevertheless no specific mutations have been reported [11]. Further advances in molecular biology of ONB may help us better understand this disease and predict the prognosis.

The initial clinical symptoms of these ONB patients were non-specific compared with chronic rhinosinusitis or other benign nasal tumors. In our study, 76.9% (10/13) had unilateral nasal obstructions, 46.2% (6/13) had repeated epistaxis and 23.1% (3/13) had visual disturbance. Minor manifestations are anosmia, headache, facial pain and epiphora. In the previous literature, the most common symptoms of patients with this tumor may include nasal obstruction, anosmia, epistaxis, and headache [1], which is consistent with the results of this study. Since most of these symptoms are nonspecific, it is difficult to make an early diagnosis which is rather vital to patients’ prognosis. A coronal CT scan is the recommended initial radiological exam and essential for staging [12]. ONB often displays a homogeneous soft-tissue mass in the upper nasal cavity, with various degrees of bony erosion. MRI scans are of value in delineating the tumor mass, presenting a low- intense signal in T1-weighted images and an iso- or high- intense signal in T2-weighted images. In our study, both imaging tests were necessary for all patients. PET-CT acts as a complementary exam, which is useful for the detection of cervical or distinct metastases.

The key to early diagnosis of ONB is an early intranasal biopsy. Typical light microscopy features of H-E staining consist of nest-like homogeneous small cells with uniform round nuclei, with rosette or pseudorosette formation, and eosinophilic fibrillary intercellular background material [6], as shown in Fig 3A, 3B and 3C. The neoplastic cells have prominent nuclei, and the cell borders are difficult to see. When the tumor tissue is undifferentiated, showing anaplastic pyknotic cell nucleus and many pathological mitotic figures, it is difficult to distinguish ONB from other small cell malignant tumors, including malignant lymphoma, Ewing tumor, malignant melanoma, and, in particular, sinonasal undifferentiated carcinoma by light microscopy [13]. In these instances, immunohistochemistry (IHC) staining is of great value. There is no specific marker for ONBs, but most of them are positive for neuron specific enolase (NSE), S-100 protein, Ki-67, synaptophysin, chromogranin and neurofilament; while negative for cytokeratin, vimentin and leucocytic common antigen (LCA). S-100 positive cells are usually found at the periphery of the lobules and correspond to sustentacular cells. These same peripheral cells may be positive with glial filament acidic protein (GFAP) [14]. The most commonly used proliferation index is Ki-67.A former study showed that longer survival rates are related significantly to a low (< 10%) Ki-67 labeling index [15], which is consistent with our study. In our thirteen cases, the highest Ki-67 labeling index was 60% (as shown in Fig 3D) and the patient only survived 23 months after treatment with the shortest follow-up.

According to the degrees of lobular architecture, mitotic index, nuclear pleomorphism, fibrillary matrix, rosettes or pseudo-rosettes, calcifications and necrosis, Hyams proposed the only histological grading system for ONB in 1988 [14]. Tumors are separated into four grades, ranging from Grade I (well differentiated) to Grade IV (least differentiated). It is complex to use this classification system due to the semi-quantitative nature and some level of discordance among pathologists. Moreover, diagnoses based on biopsies can result in sampling errors [16]. Therefore, many studies tend to divide ONB into low-grade (Hyams Ⅰ/ Ⅱ) and high-grade (Hyams Ⅲ/ Ⅳ). Malouf et al[17] drew a conclusion that patients with high-grade ONB had higher T4 stage, frequent lymph node involvement and mainly leptomeningeal metastasis in contrast to low-grade ONB. Thus, a High-grade of Hyams system portends a worse prognosis [18]. In our study, low-grade had a higher 5-year overall survival of 66.7% compared to high-grade of 28.6%. However, our study showed no statistical difference by log-rank test (P = 0.141), probably due to the small number of sample size.

Another widely used classification system is the TNM system proposed by Dulguerov in 1992 [5], based on pre-treatment CT and MRI findings. The detailed description of this system is as follows. T1: Tumor involving the nasal cavity and /or paranasal sinuses (excluding sphenold), sparing the most superior ethmoidal cells; T2: Tumor involving the nasal cavity and /or paranasal sinuses (including sphenold),with extension to or erosion of the cribriform plate; T3:Tumor extending into the orbit or protruding into the anterior cranial fossa; T4:Tumor involving the brain. N0: No cervical lymph node metastasis; N1: Any form of cervical lymph node metastasis. M0: No metastasis; M1: Distant metastases. Bachar G et al. [19] and Zafereo et al. [20] both demonstrated that TNM system was correlated most closely to survival and recurrence compared with Hymas grading and Kadish staging. In this research, the incidence of cervical involvement was 23.1% and distant metastasis was 15.4% at the time of diagnosis, within the range of 10 to 33% and 12 to 25%, reported by Castelnuovo [21]. Our study revealed that TNM staging was an important guide for prognosis. T, N and M stages were related prognostic factors with P = 0.028, 0.000, 0.007 (log-rank test), respectively, in agreement with the previous literature.

In addition, modified Kadish staging system is the most accepted staging system, although it was initially described based on 17 patients only. Stage A: Tumor limited to the nasal fossa. Stage B: Tumor extension into the paranasal sinuses. Stage C: Tumor extension beyond the paranasal sinuses and nasal cavity. Stage D: Metastases are identified in cervical lymph nodes or distant sites [3, 4]. 30.8% of patients recruited in our study were at low-stage (A/B) and 69.2% at high-stage (C/D), which showed the difficulty of early diagnosis. High-stages of modified Kadish system ended up with a significantly poorer prognosis compared to low-stage (log-rank P = 0.025), as reported in numerous literatures [8, 9].

The optimal treatment of ONB is diversified and controversial. Craniofacial resection has been considered the gold standard surgical treatment for sinonasal tumors. However, it is a radical approach with a high level of mortality and a long recovery time [22]. With the development of endoscopic approaches over the last two decades, many centers are in favor of endoscopic endonasal surgery (EES) over traditional open craniofacial surgery for ONB [23]. A meta-analysis in 2012 compared oncologic outcomes between open and endoscopic surgery, then supported the use of endoscopic approaches, citing comparable survival to open approach [24]. Clinical experiences of UCLA revealed that the endoscopic approach had a statistically significant decrease in length of hospital stay and a trend towards reduced blood loss and complications [25]. Lund VJ and colleagues summarized an eighteen-year experience and reported that endoscopic resection is an alternative to conventional craniofacial resection [26]. Based on the favorable results observed so far, the combination of endoscopic sinus surgery and radiotherapy can be considered as a promising new option for the treatment of ONB that is worthy of further investigation [27]. Researchers in Anderson Cancer Center drew a conclusion that survival is improved considerably when surgical resection is followed by postoperative radiation [28]. If the tumor tends to involve the adjacent structures which are important, such as infraorbital canal, optic nerve and optic intersection, researchers recommend intensity modulated radiation therapy (IMRT) instead of conventional radiotherapy because IMRT better preserves closer structures [29]. In addition to surgery and radiotherapy, systemic therapy also includes chemotherapy, which may offer improvement of local control and reduction of the frequency of distant metastasis, especially for patients with unresectable tumors [30].A retrospective review at the Mayo Clinic showed a possible benefit in chemotherapy with a combination of cisplatin and etoposide in high grade tumor [31]. Different institutions reveal that high grade tumors were sensitive to cisplatin-based chemotherapy. In this regard, grading has been suggested as an important factor influencing treatment decision in ONB [32]. In our study, 76.9% patients (10/13) underwent comprehensive treatment including EES while 23.1% (3/13) received radiotherapy and chemotherapy without EES. Oncologic outcomes had a significant difference between with- and without-surgery group (log-rank P = 0.015). Our experiences are summarized here. Surgery alone is effective for low-stage tumors with clear margins. Radiotherapy is used for residual lesions and for all high-stage tumors. Tumor shrinkage after neoadjuvant radiotherapy or chemotherapy can transform the case from inoperable to operable. As for extensive metastatic lesions, systemic chemotherapy and palliative radiation to local site are advised, aiming to improve the quality of life. Moreover, elective cervical dissection is carried out when preopreative examination had confirmed the existence. With regard to dosage of radiotherapy, we adopt 55 Gy before surgery and 60 Gy after surgery. The preferred chemotherapy regimen is cisplatin and etoposide, which is the same as in the Mayo Clinic [33]. It is emphasized in earlier literatures that recurrence can occur years after the completion of treatment, even after the 10-year mark. Therefore, long-term follow-up is needed [34].

The present study was subject to certain limitations. It was a retrospective case series from single institution and the number of definite diagnosed patients was limited, which could bias the results. These biases would be addressed in the future through a prospective randomized double-blinded trial. Under the condition of a large enough sample size, it will be necessary for Cox multivariate regression analysis to eliminate confounding factors. Longer follow-up will be needed to ascertain if these findings hold true.

## Conclusions

Preoperative assessment of clinical manifestations and imaging presentations are essential to determine the treatment plan. Histopathology is rather important for diagnosis and differential diagnosis. Late stages classified by the TNM and Kadish systems indicate poorer prognosis. Comprehensive treatment modality, including endoscopic endonasal surgery achieved good long-term results. Further studies are needed to better define the role and appropriate sequence of comprehensive treatment in ONB.
